# Professional Outcomes Following The Aesthetic Society–Endorsed Aesthetic Surgery Fellowships: A Single Program Fellowship Review and National Survey

**DOI:** 10.1093/asjof/ojag048

**Published:** 2026-03-18

**Authors:** Bhavana Thota, Carolyn Kim, Shikhar Tomur, Jennifer Barillas, William P Adams, Jeffrey M Kenkel

## Abstract

**Background:**

The number of The Aesthetic Society–endorsed aesthetic surgery fellowship (ASEAF) programs has grown in recent years as more plastic surgery residents pursue advanced training in aesthetic procedures following residency.

**Objectives:**

The aim of this study was to describe the structure and case volume of one long-established ASEAF program and summarize practice patterns and self-reported experiences of nationwide ASEAF graduates (from the US programs) across 6 academic years.

**Methods:**

A retrospective review of case logs from 6 aesthetic surgery fellows at a single institution from the 2018 to 2023 academic years was conducted. Additionally, an anonymous 5-question survey was distributed to 125 ASEAF graduates nationwide from the same time period.

**Results:**

Fellows at the study institution completed a total of 3113 aesthetic cases with an average distribution of 80.5% surgical and 19.5% nonsurgical procedures. Breast augmentation was the most common surgical procedure early in the study period, whereas rhinoplasty predominated in later years. Neuromodulator injections were the most common nonsurgical procedure performed. Among 64 survey respondents (51.2% response rate) representing 24 endorsed fellowship programs nationwide, most reported practicing in group private practice settings (*n* = 44, 68.8%). Facial aesthetic surgery represented the largest portion of respondents’ current practice (28%).

**Conclusions:**

This study provides descriptive insights into the structure and case volume of 1 ASEAF program and reports trends in practice settings and procedural focus among ASEAF graduates nationwide. These findings offer a snapshot of aesthetic fellowship training and early career patterns but do not assess patient outcomes or compare endorsed fellowships with other training pathways. Future research with larger cohorts, longer follow-ups, and objective measures is needed to better understand the long-term professional impact of aesthetic fellowship training.

**Level of Evidence: 5 (Therapeutic):**

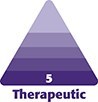

Plastic surgery residency training requires the completion of a minimum number of operative cases across all plastic surgery subspecialties per the Accreditation Council for Graduate Medical Education (ACGME; Chicago, IL).^1^ After residency, graduates can pursue further specialization in aesthetic, burn, craniofacial, gender-affirming, hand, and/or microsurgery. Between 2018 and 2022, the majority of integrated residency graduates (70%) and nearly half of independent graduates (49%) pursued fellowship training.^2^ Of those who pursued fellowship between 2013 and 2022, ∼29% selected aesthetic surgery.^3^

Recent studies indicate that plastic surgery residents feel they receive inadequate exposure to aesthetic surgery during training.^4-8^ One survey found that 53.7% of respondents felt they received the least training in aesthetic surgery, followed by burn surgery at 45.4%.^5^ In another study focused on aesthetic training, the majority of plastic surgery residents desired additional experience in facial aesthetic procedures, particularly in rhinoplasty (77.73%), followed by facelift (59.83%), brow lift (39.30%), and blepharoplasty (38.86%).^9^ These perceptions persisted despite ACGME minimum requirements intended to standardize aesthetic training, which mandate at least 150 aesthetic surgical procedures (including 30 breast, 50 head and neck, and 50 trunk and extremity), 10 laser-related procedures, and 21 injectable treatments during plastic surgery residency.^10^

Previous research has explored aspects of aesthetic surgery fellowships, including selection criteria, program trends, and applicant considerations.^11-13^ However, limited data exist on the professional outcomes of trainees who completed an aesthetic surgical fellowship endorsed by The Aesthetic Society (Garden Grove, CA). This study aims to describe the structure of one such The Aesthetic Society–endorsed aesthetic surgery fellowship (ASEAF) program and examine the experiences and career trajectories of ASEAF graduates nationwide from the 2018 to 2023 academic years.

## METHODS

This study included a single-institution retrospective review of case volumes for 6 aesthetic surgery fellows and was conducted with approval from the IRB. Case logs from the 2018 to 2023 academic years (July 1, 2018 to June 30, 2024) were analyzed. Case type and number were recorded for each fellow. Case logs included all cases in which the aesthetic fellow participated, and faculty members were present for all procedures; however, the specific degree of fellow operative involvement was not recorded.

Additionally, a broader nationwide survey was conducted for all graduates from ASEAF programs during the same time period. In February 2025, an anonymous 5-question survey was developed using Qualtrics (Provo, UT) and distributed electronically by the senior author to all 125 ASEAF graduates from the 2018 to 2023 academic years. No incentives were offered to respondents for completion of the survey. Survey items assessed fellowship graduates’ current practice setting, breadth of practice, and perceived impact of fellowship training (Table 1). Questions included multiple-choice, percentage slider, and short-answer formats. Descriptive statistics were performed using Microsoft Excel (version 16.77; Redmond, WA). Thematic analysis of the short-answer questions was performed by the entire study team. One team member conducted the initial coding, after which themes were generated collaboratively and finalized through group consensus.

**Table 1.** Survey Questions Sent to All The Aesthetic Society–endorsed Aesthetic Surgery Fellowship Graduates During the 2018-2023 Academic Years

Question type	Question
Multiple choice	Which of the following describes your current practice setting: private practice group, private practice solo, academic/university, or other?
Percentage slider	Please list the percentage (0%-100%) of your current practice that each of the following constitutes: aesthetic face, aesthetic breast, aesthetic body, nonsurgical treatments, reconstructive, and other.
Short answer	Please describe what you consider to be some of your biggest successes since coming out of aesthetic surgery fellowship.
Short answer	Please describe what you consider to have been the most valuable part of the aesthetic surgery fellowship.
Short answer	At which institution did you complete your aesthetic surgery fellowship?

## RESULTS

### Single-Institution Retrospective Review

**Figure 1.**
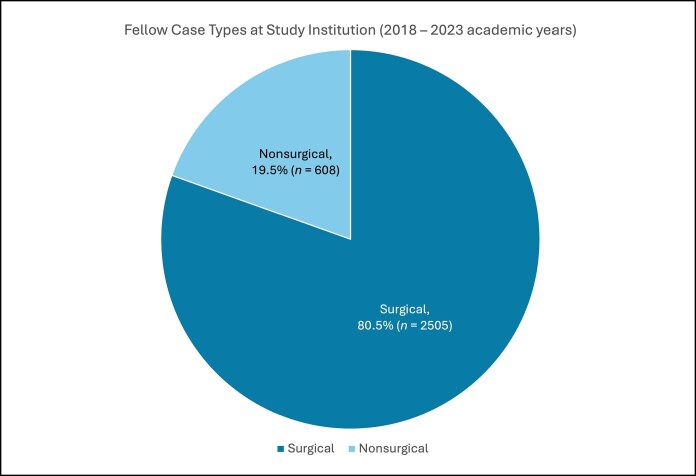
Surgical vs nonsurgical aesthetic fellow cases at the study institution during the 2018-2023 academic years.

**Figure 2.**
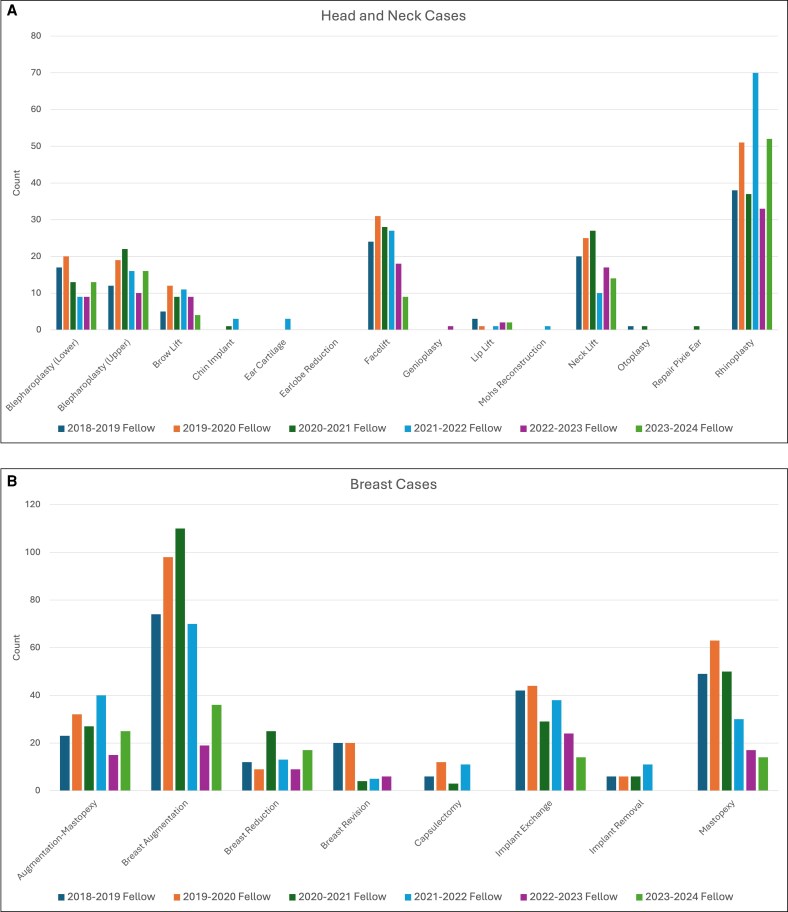

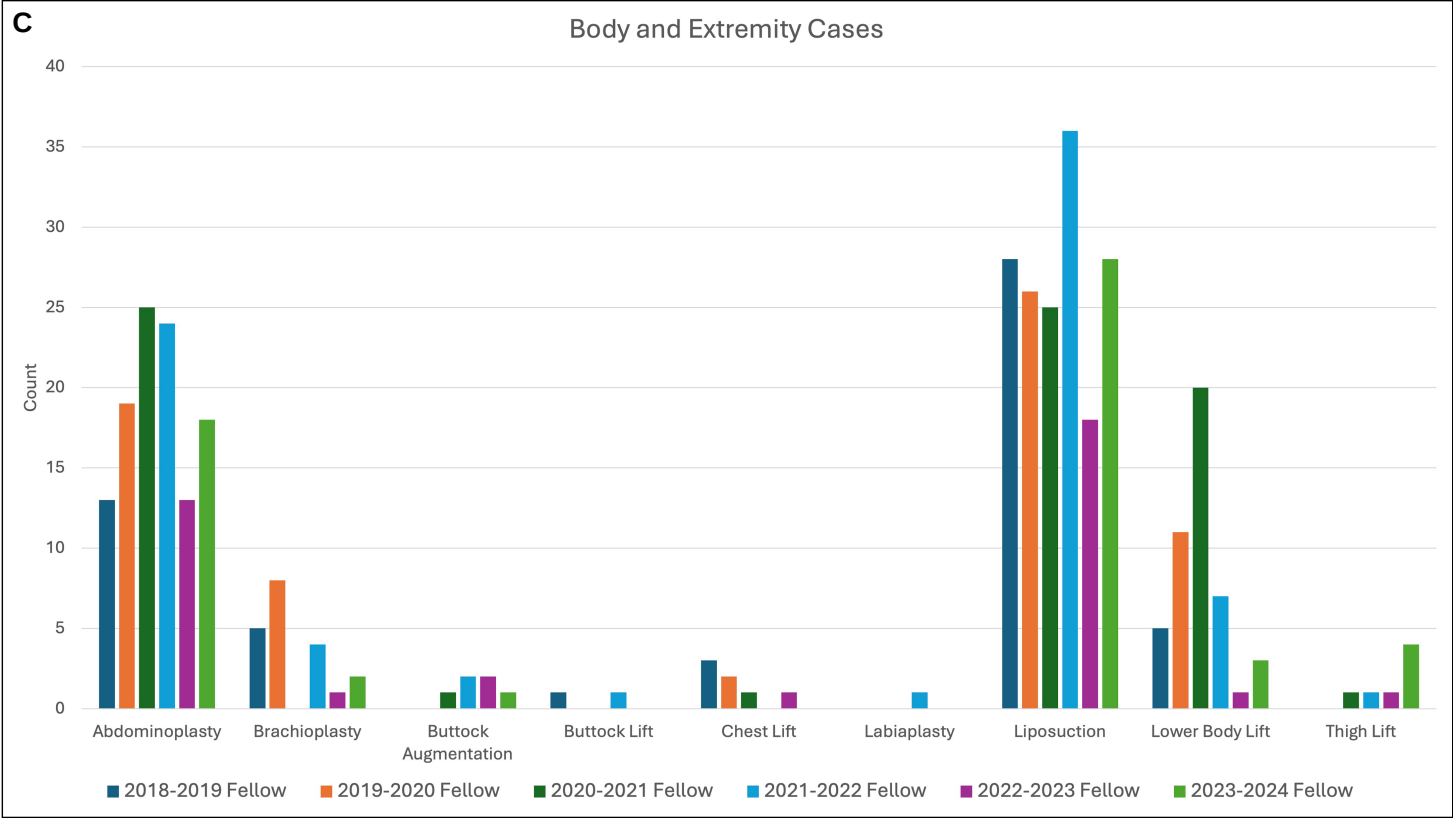
Breakdown of surgical cases by (A) head and neck, (B) breast, and (C) body and extremity performed by each aesthetic fellow at the study institution during the 2018-2023 academic years.

**Figure 3.**
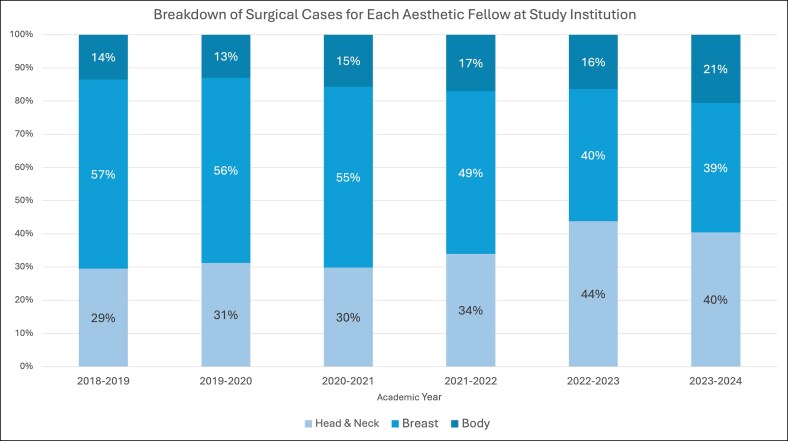
Percentages of surgical case types performed by each aesthetic fellow at the study institution during the 2018-2023 academic years.

**Figure 4.**
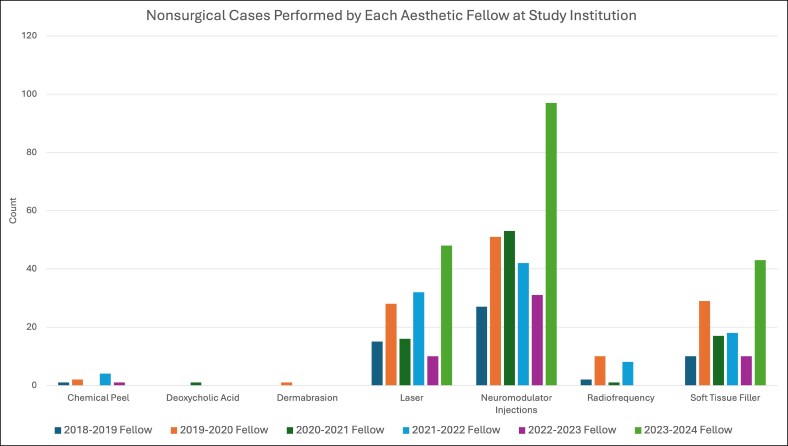
Breakdown of nonsurgical cases by each aesthetic fellow at the study institution during the 2018-2023 academic years.

The 6 aesthetic surgery fellows who completed the study institution's ASEAF program between the 2018 and 2023 academic years collectively performed a total of 3113 aesthetic cases, with a median of 523 cases per fellow (standard deviation 109.8). On average, ∼80% of fellow cases were surgical (*n* = 2505) and 20% nonsurgical (*n* = 608), including soft tissue fillers, neuromodulator injections, and laser or energy-based treatments (Figure 1). The most common surgical procedure was breast augmentation for the first 3 fellows, equal numbers of breast augmentations and rhinoplasties for the fourth fellow, and rhinoplasty for the last 2 fellows (Figure 2). This trend corresponded with an increasing proportion of head and neck procedures over time (Figure 3). Neuromodulator injections were the most frequently performed nonsurgical procedure across all 6 academic years (Figure 4).

### Nationwide Survey

**Figure 5.**
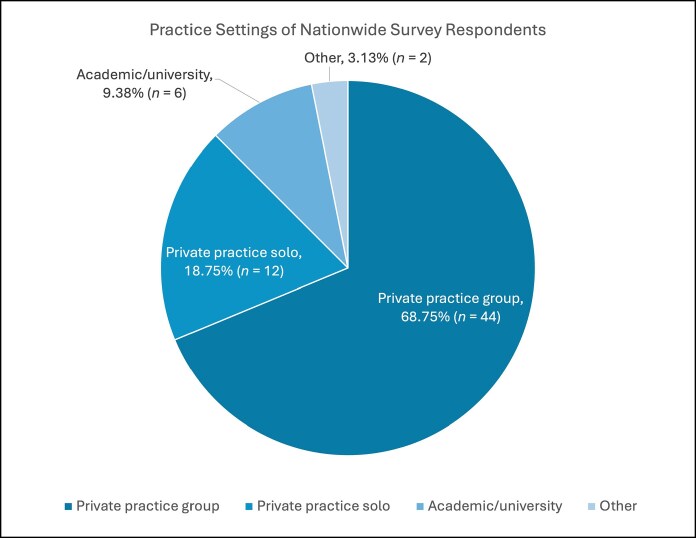
Practice settings of nationwide The Aesthetic Society–endorsed aesthetic surgery fellowship graduates from the 2018-2023 academic years.

**Figure 6.**
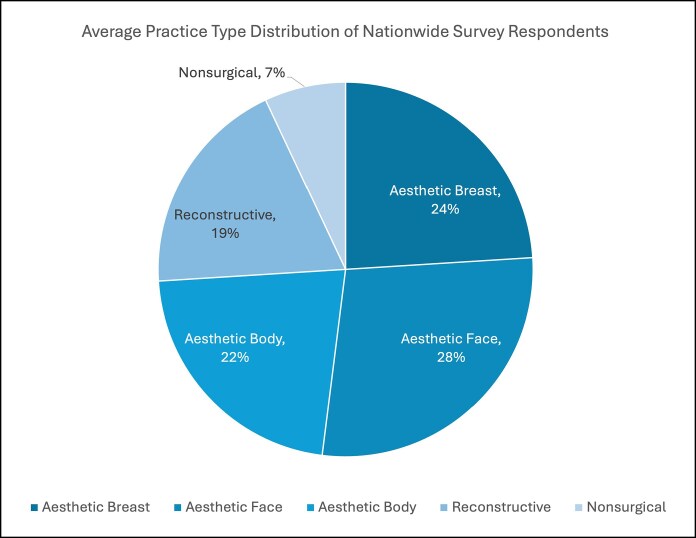
Average practice type distribution of nationwide The Aesthetic Society–endorsed aesthetic surgery fellowship graduates from the 2018-2023 academic years.

Of the 125 ASEAF graduates surveyed, 64 responded (51.2%), representing 24 programs across the United States. At the time of survey dissemination, most respondents practiced in group private practice settings (*n* = 44, 68.8%; Figure 5). Two respondents who listed “other” reported mixed academic and private practice or solo private practice licensed in a group. On average, the largest portion of respondents’ practices was facial aesthetics (28%; Figure 6).

Responses to both short-answer questions regarding successes following completion of fellowship and the most valuable part of aesthetic fellowship were largely positive (*n* = 120 responses, 96.8%). Responses included repeat language across those who completed the survey. Commonly reported successes following fellowship included securing competitive positions (*n* = 30, 48.4%), increased confidence (*n* = 14, 22.6%), and enhanced knowledge/skills (*n* = 12, 19.4%; Supplemental Table 1). Additional themes included improved patient outcomes, board certification, and networking. When asked about the most valuable aspect of aesthetic surgery fellowship, respondents cited skill refinement (*n* = 21, 33.9%), confidence building (*n* = 18, 29.0%), and high case volume (*n* = 13, 21.0%; Supplemental Table 2). Other notable responses included mentorship, networking with local aesthetic plastic surgeons, specialized facial aesthetics training, and practice management.

## DISCUSSION

The first aesthetic surgery fellowship was established in 1970 to address the lack of such specialized training at that time.^14^ Since then, the number of endorsed aesthetic surgery fellowships has grown substantially, particularly after joining the San Francisco match in the 2017-2018 cycle. In 2018, there were 14 endorsed fellowship programs; by 2025, this number has increased to 38.^15,16^ This expansion parallels a broader trend toward subspecialization in plastic surgery, with microsurgery and aesthetic fellowships showing the greatest growth in number of applicants between 2013 and 2022.^3^ With the growing number of aesthetic surgery fellowship positions and applicants, understanding the structure and impact of ASEAF programs is critical. To our knowledge, this is the first study to describe the specific components of a single ASEAF program and examine the experiences and career trajectories of ASEAF graduates nationwide.

Like many fellowships around the country, the aesthetic surgery fellowship at the study institution has been in existence for >20 years and offers a broad exposure of the subspecialty through a 1-year experience. Endorsement by The Aesthetic Society requires programs to adhere to specific criteria. These include a comprehensive aesthetic curriculum, independent clinic and operative experience, and mentorship on the business side of plastic surgery, such as clinic management, aesthetic consultation, and practice development.^17^ Affiliation with an ACGME-approved program further helps facilitate and stimulate scholarly activity. This may include lectures, roundtable discussions, and research opportunities. Through structured dialogue both inside and outside of the operating room, endorsed fellowships deliver training that extends beyond technical proficiency to encompass the nuances of aesthetic practice.

Although these core requirements provide a framework, they allow flexibility based on the patient population and procedures offered within a given institution.^18^ Studies indicate that fellows and program directors prioritize broad surgical exposure and autonomy.^18^ Consistent with previous literature, plastic surgery residents reported lower confidence in head and neck aesthetic procedures compared with breast or trunk/extremity procedures.^9,19-21^ In particular, the majority of residents noted rhinoplasty as the number one procedure they would like to receive further training on.^9^ Our findings echo this sentiment as nationwide survey respondents cited facial aesthetic training among the most valuable aspects of fellowship.

Survey results also suggest that fellowship training may influence practice composition. Nationwide ASEAF graduates reported that facial aesthetics (28%) constituted the largest portion of their practices, followed by breast aesthetics (24%) and body aesthetics (22%; Figure 6). Short-answer responses emphasized increased confidence and skill refinement in facial procedures, reinforcing the importance of this training focus.

Dedicated aesthetic fellowship training appears to be considered a positive factor when hiring an aesthetic plastic surgeon.^18^ ASEAF graduates nationwide noted their fellowship experience was instrumental in securing competitive positions, most commonly within established group private practice settings (68.8%). Additionally, most ASEAF programs are located in large metropolitan areas, allowing for not only a larger referral base and high case volumes but also more robust networking and mentorship opportunities, which graduates identified as beneficial for both job placement and career clarity.^16^

This study has several limitations. First, the single-institution review may not reflect the full range of experiences across ASEAF programs nationally. Additionally, the fellow case logs did not document the degree of operative involvement, limiting conclusions about operative independence; however, our aim in evaluating this data was to characterize fellow case volume and variety rather than to assess autonomy. Second, the nationwide survey had a modest 51.2% response rate, which may introduce nonresponse bias and limits generalizability. The survey's short-answer questions were not validated and relied on anecdotal, self-reported perceptions of satisfaction and training value, which are inherently subjective and susceptible to response bias. Although thematic analysis was performed by the entire study team, the evaluators were affiliated with an endorsed aesthetic fellowship program, and the possibility of confirmation bias cannot be fully excluded. Third, the absence of a comparison group who did not complete an endorsed fellowship prevents direct assessment of the differential impact of ASEAF training on career outcomes. Finally, the graduates included in this study are, at most, 6 years post fellowship, which may be insufficient time to capture the long-term professional achievements or the full trajectory of practice development. Future studies with larger cohorts and extended follow-ups may consider incorporating additional metrics, such as professional society memberships, leadership positions, and academic or industry awards or recognition.

## CONCLUSIONS

Aesthetic surgery is an integral and complex subspecialty that often requires additional focused training beyond plastic surgery residency, particularly for surgeons who plan to make aesthetic surgery a cornerstone of their practice. This study provides descriptive insights into the structure and case volume of a long-established ASEAF program and shares survey responses from ASEAF graduates nationwide across 6 academic years (2018-2023). Our findings characterize common practice settings, focus areas, and self-reported reflections on fellowship experiences, offering a snapshot of current trends in aesthetic fellowship training and early career trajectories. However, these results do not assess patient outcomes or compare effectiveness between endorsed and nonendorsed fellowships. Future research should incorporate larger cohorts, extended follow-ups, and objective measures, such as patient outcomes to better understand the long-term professional impact of aesthetic fellowship training.

## Supplementary Material

ojag048_Supplementary_Data
