# Supplementary material for: Professional Outcomes Following The Aesthetic Society–Endorsed Aesthetic Surgery Fellowships: A Single Program Fellowship Review and National Survey
Source: Aesthet Surg J Open Forum. 2026 Mar 18;8:ojag048. doi: 10.1093/asjof/ojag048 (PMC13089856; doi:10.1093/asjof/ojag048)
Supplement: ojag048_Supplementary_Data [file ojag048_supplementary_data.zip › Supplemental Table 1.docx]

**Supplemental Table 1**. Short-answer Responses Describing Successes Following Graduation From an Aesthetic Society-endorsed Aesthetic Fellowship Program

| Short-answer responses | Themes |
| --- | --- |
| Increased confidence with rhinoplasty and facelift. | Confidence |
| I have quickly been able to establish myself as an expert in aesthetic surgery despite my limited time in practice. This is entirely due to my training in fellowship. | Knowledge/Skills |
| An advanced understanding of aesthetic surgery/anatomy/indications/complication management. | Knowledge/Skills |
| Aesthetic surgery is hard, no successes yet. | NA |
| Securing a position in an aesthetic only private practice. | Job |
| Getting out of academia. | Job |
| Performing successfully and timely a deep plane facelift with fat grafting and laser resurfacing. | Knowledge/Skills |
| Being able to jump right into 100% aesthetic practice, month over month volume growth. | Job |
| The opportunity to do mostly aesthetic surgery, early on. | Job |
| Complete comfort with bread and butter aesthetic surgery. | Confidence |
| Confidence, results, mentorship, and relationship with colleagues to bounce ideas and difficult cases off of. | Confidence |
| Building a practice of my own. | Job |
| Comfortable performing facial aesthetic cases. | Confidence |
| Buying into and eventually taking over a solo practice. | Job |
| Competent in executing all cosmetic techniques from day 1, allowing me to obtain good results and build my practice faster than I would if I had undertaken done a fellowship. | Outcomes |
| Negotiating and executing a purchase opportunity with the aesthetic practice that I joined. | Job |
| Starting and running my own practice and providing livelihood for my employees. | Job |
| Great patient outcomes. | Outcomes |
| Facial aesthetics practice, ASERF grant. | Job |
| Building a solo practice. | Job |
| Better understanding of breast and body aesthetics; passing boards. | Knowledge/Skills; Board certification |
| Feeling very comfortable offering a wide range of aesthetic procedures in my first year in practice. | Confidence |
| That I will treat patients, colleagues, people better than how they were treated in fellowship. That I never needed an aesthetics fellowship and doing one was the biggest regret I’ve made career wise and ethically. I did learn I will never sacrifice my ethics to make money at the expense of my patients. | NA |
| Getting the perfect job! | Job |
| Joining an 100% aesthetic surgery practice, becoming program director for our ASAPS-endorsed fellowship, being involved in device studies. | Job |
| Confidence with patient consultation, surgical selection and execution. | Confidence |
| Working for a practice that is 100% cosmetic. | Job |
| Building on the concepts learned in fellowship. | Knowledge/Skills |
| Obtaining ABPS board certification, joining ACPS as an associate, developing a busy aesthetic practice quickly without having to take ER call and without having to accept insurance cases. | Board certification; Job |
| Able to perform facial procedures well. | Knowledge/Skills |
| Board Certification within my first year out in practice; True comfort in doing revision surgery to the point I am a referral center with a practice focused 40% on cosmetic revision surgery. | Board certification; Confidence |
| Being able to immediately carve out a niche in private practice. | Job |
| Building a strong mommy makeover practice. | Job |
| Joining group practice that I trust and a place where I can continue to learn. | Job |
| The knowledge and expertise, the surgical skills, and the ability to perform aesthetic surgery techniques immediately after fellowship. | Knowledge/Skills |
| Becoming medical director and then chief medical officer of the practice. Stepping into a legacy practice. | Job |
| Being able to open a solo practice within my first 2 years out of training that is profitable (started my practice 4 months ago). | Job |
| Ability to start cases from day one with more confidence. | Confidence |
| Rapidly establishing my aesthetic surgical practice and successfully and confidently being able to integrate advanced aesthetic surgical techniques into my practice. | Job; Confidence |
| I have been able to do incredibly challenging breast and body revision cases, which is only possible thanks to my fellowship. Additionally, I am on track to reach the 50 cases needed to be eligible for oral boards which has seemed to be a struggle for many this year. | Knowledge/Skills; Board certification |
| Networking with other surgeons from the same area, started booking and performing my own surgeries. | Networking |
| Opening my own practice. | Job |
| Building my own 100% aesthetic practice. | Job |
| Receiving positive patient feedback and reviews. | Outcomes |
| More confidence in doing procedures that weren’t taught or had I had little experience to in residency. | Confidence |
| Starting a successful solo private practice in a competitive market. | Job |
| Complex breast revisions. | Knowledge/Skills |
| Securing a job with a well-established practice in a highly competitive market. | Job |
| Having the confidence to start solo practice. | Job |
| Starting solo practice in a major city. | Job |
| Good job. | Job |
| Collecting cases, Passing boards | Board certification |
| Confidence in taking on tough cases and revision cases. Ability to start a near aesthetic only practice right out of fellowship. | Confidence; Job |
| Still working hard to become successful. | NA |
| Confidence with high level aesthetic procedures: deep plane face/neck rejuvenation, advanced revision cosmetic breast | Confidence |
| Applying concepts learned in fellowship. | Knowledge/Skills |
| Growing my patient base. | Job |
| Comprehensive understanding of facial aging and all the state of the art surgical techniques and exposure to nearly every nonsurgical device/product on the market. | Knowledge/Skills |
| The medical knowledge, surgical ability, and years of training/experience that you achieve from the moment you complete fellowship. | Knowledge/Skills |
| My fellowship set me up SO well to get started in full time aesthetic practice. My biggest success would be establishing myself as a provider of facial aesthetic procedures in a busy market with 1 full OR day each week. | Job |
| Feeling much more comfortable making and executing my surgical plan for aesthetic patients. Performing multiple aesthetic operations at one time. | Confidence |
| Comfort level and outcomes with complicated rhinoplasty. | Confidence |
