# Supplementary material for: Professional Outcomes Following The Aesthetic Society–Endorsed Aesthetic Surgery Fellowships: A Single Program Fellowship Review and National Survey
Source: Aesthet Surg J Open Forum. 2026 Mar 18;8:ojag048. doi: 10.1093/asjof/ojag048 (PMC13089856; doi:10.1093/asjof/ojag048)
Supplement: ojag048_Supplementary_Data [file ojag048_supplementary_data.zip › Supplemental Table 2.docx]

**Supplemental Table 2**. Short-answer Responses Describing the Most Valuable Parts of Aesthetic Society-endorsed Aesthetic Fellowship Programs

| Short-answer responses | Themes |
| --- | --- |
| Rhinoplasty experience. | Facial aesthetics |
| The clinical training I received was invaluable. It has instantly made me the best trained aesthetic surgeon in my market. Also, the relationships I made in fellowship both with the attendings I worked with as well as prior fellows has been a tremendous asset. It has helped boost my position within the Aesthetic Society as well as create a network of world-class surgeons to consult on difficult cases. | Skills; Networking |
| All of it was key in my development. I don't think I could do what I am doing without having done my fellowship. | Skills |
| High repetition exposure to aesthetic procedures that is completely absent from residency training. | Skills |
| Determining the correct operation for each patient, based on their goals. Seeing multiple procedures, and subsequent outcomes in training, I have been more prepared to offer a tailored approach anticipating outcomes and matching patients with the most appropriate procedure. | Skills |
| Networking. | Networking |
| Confidence of dealing with almost any cosmetic issue, even now I cannot perform at my full capacity I have seen what a full capacity looks like and what is humanly possible in terms of skills, efficiency, productivity, teamwork. | Confidence |
| Confidence consulting with patients, experience in facelift, scope of skillset. | Confidence |
| The opportunity to perform aesthetic surgeries at a discounted rate, to rapidly become more comfortable. | Confidence |
| Learning to manage the difficult aesthetic patient psyche. | Confidence |
| Confidence, results, mentorship and relationship with colleagues to bounce ideas and difficult cases off of. | Confidence; Mentorship |
| Hands on experience, getting to feel more comfortable with the procedures. | Skills; Confidence |
| Lifelong mentorship. | Mentorship |
| In surgery technical training, refinements of techniques, business mentoring, exposure to non-surgical aesthetic modalities. | Skills; Mentorship |
| The fact that the entire year was focused on cosmetic surgery. Time spent in the OR performing (just cosmetic) surgeries alongside expert surgeons. | Skills |
| Fellowship clinic and ability to learn from and apply principles learned from experts in the field. | Skills |
| A dedicated year to focus on aesthetic surgery and mentorship from faculty you want to model your practice after. | Mentorship |
| Techniques. | Skills |
| Facial aesthetic training. | Facial aesthetics |
| Refining techniques and becoming more confident with complex aesthetic cases (revision face, revision rhinoplasty). | Skills; Confidence |
| Repetitions in breast and body surgery. Getting to do my own cases with advice from the partners in the practice was invaluable. | Case volume |
| High case volume and variety of aesthetic procedures. | Case volume |
| Realization that private practice can be extremely greedy, ethics blurred, patient care not important compared to making money. | Business |
| I didn’t know what I didn’t know and it showed me so much more than I thought it ever would. | Skills |
| Feeling comfortable performing a wide range of complex aesthetic surgeries. | Confidence |
| Facial aesthetic training (facelift/neck lift, eyes, nose). | Facial aesthetics |
| Honing skill in aesthetic surgery, learning the business side of plastic surgery. | Skills; Business |
| Hands on experience in and out of OR and seeing the behind the scenes on how to run a practice (from patients to staff to consents). | Skills; Business |
| High volume center with significant hands-on experience, fellow clinic that was very well supported (fellow clinic with dedicated patient coordinator, protected time in the OR, marketing for fellowship done by the practice). | Case volume |
| Learning true mastery of these techniques. Hit the ground running. | Skills |
| Exposure to various techniques, as well as being able to pick the brains of various surgeons in regard to post-operative management and practice patterns. | Case volume |
| Lifelong mentors to guide me through my early career. | Mentorship |
| Repetitions and seeing the same surgery done many ways in high volume. | Case volume |
| Having a Fellow Clinic. | Case volume |
| The one on one mentorship, high volume of aesthetic surgery, and surgical autonomy. | Mentorship; Case volume |
| Practice management, business management, efficiency. | Business |
| Confidence in surgical judgement and managing complications. | Confidence |
| Building confidence in performing aesthetic cases. Understanding how to perform in a private practice setting better. | Confidence |
| Exposure to and independent experience with facial aesthetic surgery. | Facial aesthetics |
| Building the confidence and the skills to be a good aesthetic surgeon right after training. It was invaluable to learn from the decades of experience that my mentors have. I learned so much about pre-op counseling and post-op complications and management, as well as practice management. | Confidence; Skills; Mentorship; Business |
| Confidence during consultation (to discuss a treatment/surgical plan) and confidence when performing surgeries. | Confidence |
| The volume of aesthetic cases I was able to perform during fellowship placed me years ahead of where I would have been from a confidence standpoint surgically. | Case volume; Confidence |
| The aesthetic surgery volume, business experience and continued mentorship. | Case volume; Business; Mentorship |
| Learning new surgical techniques and training with new devices. | Skills |
| My co-fellow. She is a close colleague that I lean on for questions and opinions all of the time. Solo private practice is like being on an island, so having her (and a few close mentors) is priceless. | Networking |
| Business aspect of the model. Getting hospital privileges. | Business |
| Repetition and being immersed in aesthetic surgery | Case volume |
| Having the framework and understanding of the private practice space to feel confident starting off in a small practice, which has historically not offered breast and body operations. | Confidence |
| Exposure to the business considerations of a plastic surgery practice. | Business |
| Better techniques/increased confidence in aesthetic procedures especially facelift, rhinoplasty, and revision procedures. | Skills; Confidence |
| Education. | Case volume |
| Repetition of similar cases. | Case volume |
| Picking up nuances of technically challenging aesthetic procedures. | Skills |
| Seeing multiple masters perform complex aesthetic procedures. | Mentorship |
| Fellow clinic for the independence, and the breadth/depth of cosmetic exposure at a high level. | Case volume |
| Learning not only surgical techniques, approaches, indications, but learning the clinic aspects of cosmetic surgery and the business of running a practice. | Business |
| Very sophisticated training in exactly what I wanted to do rather than generalized training. | Skills |
| Learning deep plane facelift and deep neck techniques and building a network of expert colleagues and friends in the neighborhood I practice in. | Skills; Networking |
| The mentorship and fast forwarding my career/ability/confidence about 5 years into practice. | Mentorship; Confidence |
| A solid foundation in complex facial aesthetic procedures (deep plane facelift, revision rhinoplasty, deep neck lift) and the confidence to keep developing these techniques. | Facial aesthetics; Confidence |
| Understanding the fundamentals of aesthetic surgery and patient selection. | Skills |
| Repetition of Aesthetic Face cases and access to masters of many disciplines. | Facial aesthetics |
